# Electrocardiographic patch devices and contemporary wireless cardiac monitoring

**DOI:** 10.3389/fphys.2015.00149

**Published:** 2015-05-27

**Authors:** Erik Fung, Marjo-Riitta Järvelin, Rahul N. Doshi, Jerold S. Shinbane, Steven K. Carlson, Luanda P. Grazette, Philip M. Chang, Rajbir S. Sangha, Heikki V. Huikuri, Nicholas S. Peters

**Affiliations:** ^1^Division of Cardiovascular Medicine, University of Southern California and Keck Medical Center of University of Southern CaliforniaLos Angeles, CA, USA; ^2^Department of Medicine, Geisel School of Medicine, Dartmouth CollegeHanover, NH, USA; ^3^Department of Epidemiology and Biostatistics, Medical Research Council Health Protection Agency Centre for Environment and Health, School of Public Health, Imperial College LondonLondon, UK; ^4^Digital Health Kitchen, Institute for Digital HealthLondon, UK; ^5^Faculty of Medicine, Center for Life Course Epidemiology, University of OuluOulu, Finland; ^6^Biocenter Oulu, University of OuluOulu, Finland; ^7^Unit of Primary Care, Oulu University HospitalOulu, Finland; ^8^Division of Cardiology, Children's Hospital Los AngelesLos Angeles, CA, USA; ^9^Section of Cardiology, Dartmouth-Hitchcock Medical CenterLebanon, NH, USA; ^10^Medical Research Center Oulu, Institute of Clinical Medicine, Oulu University Hospital and University of OuluOulu, Finland; ^11^National Heart and Lung Institute, Imperial College London, St Mary's and Hammersmith HospitalsLondon, UK

**Keywords:** electrocardiography, medical devices, arrhythmias, cardiac, conduction system disorders, ambulatory patients, healthcare delivery

## Abstract

Cardiac electrophysiologic derangements often coexist with disorders of the circulatory system. Capturing and diagnosing arrhythmias and conduction system disease may lead to a change in diagnosis, clinical management and patient outcomes. Standard 12-lead electrocardiogram (ECG), Holter monitors and event recorders have served as useful diagnostic tools over the last few decades. However, their shortcomings are only recently being addressed by emerging technologies. With advances in device miniaturization and wireless technologies, and changing consumer expectations, wearable “on-body” ECG patch devices have evolved to meet contemporary needs. These devices are unobtrusive and easy to use, leading to increased device wear time and diagnostic yield. While becoming the standard for detecting arrhythmias and conduction system disorders in the outpatient setting where continuous ECG monitoring in the short to medium term (days to weeks) is indicated, these cardiac devices and related digital mobile health technologies are reshaping the clinician-patient interface with important implications for future healthcare delivery.

## Introduction

Sustained and intermittent atrial and ventricular arrhythmias, conduction system disease, and abnormally high ectopic burden can be important markers of cardiovascular disease in the appropriate clinical settings. Their presence may also reflect underlying myocardial ischemia, inflammation, cardiac fibrosis, myocardial tissue inhomogeneity, and/or mechanical stress from deranged hemodynamics. An inappropriately high burden of ventricular premature complexes (>20,000 per day) and frequent tachyarrhythmias may result, over time, in left ventricular dysfunction and heart failure due to tachycardia-mediated cardiomyopathy (Shinbane et al., 1997; Duffee et al., 1998; Takemoto et al., 2005). Atrial arrhythmias such as atrial fibrillation (AF) and atrial flutter (AFL) are prothrombotic (Gallagher et al., 1997; Zipes, 1997; Sparks et al., 1999; Stoddard, 2000; Thambidorai et al., 2005; Alyeshmerni et al., 2013), predisposing to cardioembolic stroke (Wolf et al., 1978; Biblo et al., 2001; Halligan et al., 2004; Parikh et al., 2012). Importantly, accumulating evidence supports a link between AF and sudden cardiac death as well as increased mortality and rehospitalization related to congestive heart failure (Mentz et al., 2012; Chen et al., 2013; Reinier et al., 2014). Sustained or intermittent bradyarrhythmias can also lead to functional limitations in activity, lightheadedness, near syncope and syncope, can limit or contraindicate the use of medications for heart failure or arrhythmias, and can predispose to pause-dependent tachyarrhythmias such as torsade de pointes. Thus, early detection of, and prompt intervention on, suspect arrhythmias may prevent the subsequent development of worsening functional class and devastating complications including cardiomyopathy, heart failure, stroke and sudden cardiac death. In cardiomyopathy and heart failure, detection and control of arrhythmia can result in reversal of pathologic remodeling. Conventional and recently developed non-invasive ambulatory electrocardiographic (AECG) monitors can be useful for revealing previously undiagnosed arrhythmias of significance and may alter the course of clinical management.

**Figure 1:**
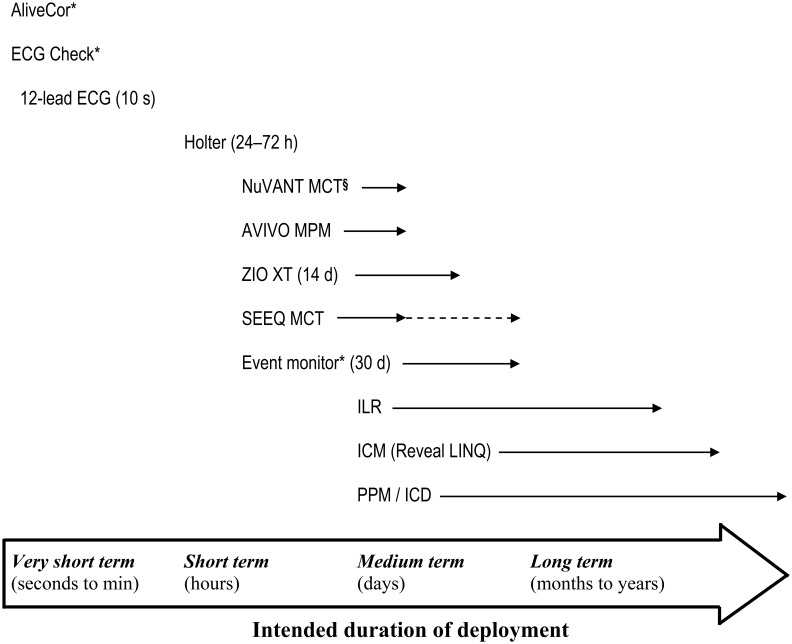
**Contemporary options for cardiac monitoring**. The range of options for outpatient cardiac monitoring varies depending on the intended study duration, the presence or absence of symptoms, the need for continuous deployment (solid line with arrows) vs. intermittent symptom-triggered monitoring, ability of the subject to activate or initiate recording, likelihood of study completion specific to device design, and lifestyle (e.g., hindrance to work and activities, need for water resistance, ability to tolerate presence of device). Dashed line with arrow indicates serial deployment of multiple patch devices to achieve a study period of 30 days. ICD, implantable cardioverter defibrillator. ICM, injectable cardiac monitor. ILR, implantable loop recorder. PPM, pacemaker. ^*^Manual contact and triggering required for intermittent activation or operation. ^§^Superseded by SEEQ MCT.

The term “cardiac arrhythmia” encompasses a spectrum of rhythm disturbances that may or may not be symptomatic. Symptoms such as palpitations, lightheadedness and syncope are important indications for performing AECG. Furthermore, cardiac studies such as echocardiography showing atrial and ventricular tachy- or bradyarrhythmias and ECG findings of conduction pathway anomalies, would also prompt a period of continuous ECG monitoring in order to investigate temporal variations, overall burden, and to enhance diagnosis. A 12-lead ECG provides a detailed, calibrated snapshot of heart rate, rhythm, conduction, and repolarization from multiple lead vectors within a 10-second time frame. Pathophysiologic states including current or pre-existing ischemia, infarction, left ventricular hypertrophy, and heritable arrhythmic disorders may also be revealed. But it is often insufficient for ruling out intermittent arrhythmias especially in the outpatient setting. The strength of a 12-lead ECG is the ability to assess rhythm, conduction, repolarization from multiple lead vectors allowing diagnosis of cardiac structural, electrophysiologic, and metabolic abnormalities and drug effects, but it is limited by the duration of rhythm detection. The choice and study duration of continuous ECG monitoring (Figure 1) is guided by the clinical questions to be answered from clinical history and physical examination. With increasing availability of ECG monitoring as consumer products, patients' preferences may also dictate the duration and mode of monitoring required and, in turn, the selection of device to use. In general, for a shorter study period (e.g., days vs. weeks to months) placement of a device that is relatively less invasive at the time of deployment (e.g., wearable monitor vs. implantable loop recorder) is preferable.

In documenting and reporting arrhythmic events, the term “episode” is used to describe onset of a self-limiting bout of arrhythmia and an episode count can be useful for paroxysmal arrhythmic events or salvos; however, the descriptor lacks a temporal dimension and does not incorporate frequency and duration of the arrhythmia. “Arrhythmia burden” can be defined as the percentage of time in arrhythmia over the total interval of recording (Euler and Friedman, 2003). This definition has been accepted as the standard for quantifying the overall frequency or extent of arrhythmic episodes, and serves also as a durable surrogate endpoint for clinical outcomes in demonstrating biologic effects of a therapy on the target arrhythmia (Euler and Friedman, 2003). For instance, successful suppression of arrhythmia by an antiarrhythmic drug should result in a reduction in arrhythmia burden.

This review summarizes not only the options and contemporary use of AECG and related devices but also highlights the changing consumer access to increasingly ubiquitous mobile medical devices, consumers' growing involvement in self-care and diagnosis, and the implications for personal controlled health records (PCHR).

## Trends in ambulatory ECG and rhythm monitoring systems

**Figure 2:**
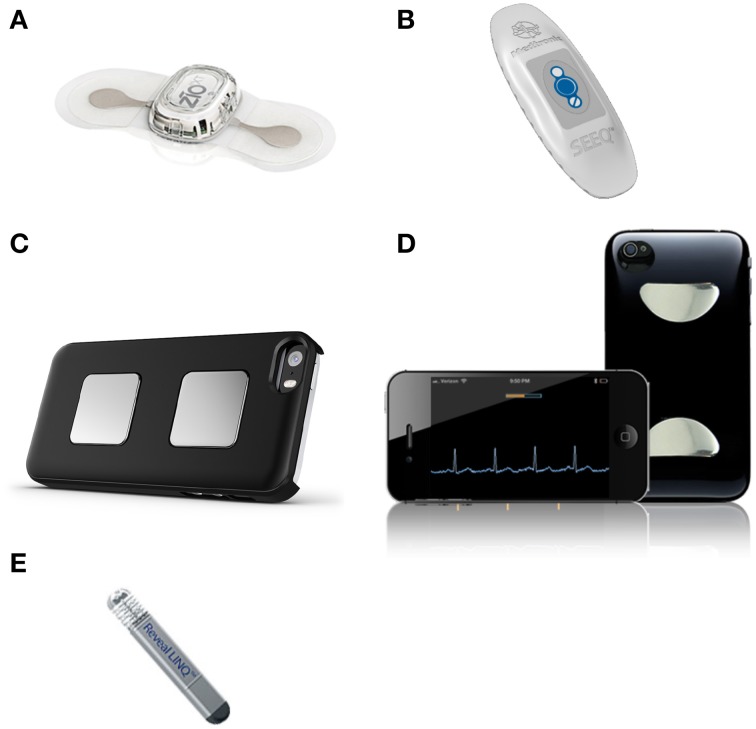
**A selection of contemporary wireless mobile cardiac monitoring devices**. Two leading AECG adhesive patch devices on the medical device market today are **(A)** second-generation ZIO® XT Patch by iRhythm Technologies, Inc. and **(B)** SEEQ™ MCT patch device by Medtronic, Inc. (cellular transmitter not shown). Featuring touch-activable electrodes configured for the Apple iPhone or Androidbased systems are **(C)** third-generation AliveCor® by AliveCor, Inc. and **(D)** ECG Check by Cardiac Designs, LLC. As the first-in-class injectable cardiac monitor, **(E)** Reveal LINQ™ (4.0 × 7.2 × 44.8 mm; 2.4 g) by Medtronic, Inc. can record rhythm data for up to 3 years.

In recent years, innovative engineering and advances in manufacturing have hastened the development of miniaturized medical devices, and yielded a variety of cardiac monitors for ambulatory use. These recently developed wearable, “on-body” ambulatory devices have integrated microelectronics (e.g., ZIO® XT Patch, NUVANT® or SEEQ® MCT; see below) for short to medium term (days to weeks) monitoring, and are challenging conventional, widely used devices from the last decades that were limited to wearable multi-lead 24-/48-h Holter monitors and event recorders (Figure 1 and Table 1). Further on the pioneering front, very short-term (seconds to minutes) handheld smartphone-enabled systems (e.g., AliveCor®, ECG Check) (Figure 2) are beginning to reshape the field of mobile cardiac monitors as well as the clinician-patient interface. These systems require attachment of an electrode-embedded module to a smartphone that detects electrical impulses from the user's fingertips and transmits signals to the mobile device to generate continuous single-channel ECG for the duration of the contact between the fingers and the sensor. While the open commercial availability of these handheld devices reduces barriers and access to health technology, health insurance plans in North America currently regard their use as experimental or investigational with no pre-approval for reimbursement. Consumers' ability to self-perform continuous event recording without a physician's input is a paradigm shift with both opportunity due to the ubiquitous presence and use of mobile devices around the world, and challenge regarding appropriate and timely interpretation of arrhythmic events. With time, validation and increased acceptance, the above smartphone-enabled devices will likely have a defined indication in outpatient heart rhythm monitoring.

**Table 1:** **Comparison between two CE-marked, FDA-approved leadless, continuous electrocardiographic patch devices and the standard Holter monitor**.

	**ZIO® XT**	**SEEQ™ (formerly NUVANT) MCT**	**Holter monitor**
Manufacturer	iRhythm Technologies, Inc.	Medtronic, Inc. (Corventis)^*^	Variable
Data storage capacity	14 days	7.5 days; up to 30 days with deployment of multiple units	24–72 h
Method of application	Timed adhesive	Timed adhesive	Multiple detachable leads and adhesive pads
Number of ECG channel(s)	1	1	Multiple (typically, 3 and up to 12)
ECG resolution (bits)	10	16	Variable
ECG sample rate (Hz)	200	200	Variable
Detection range of heart rate (bpm)	0 to >300	25–250	Variable
Symptom trigger	Yes	Yes	Yes
Water resistant	Yes	Yes	No
Data transmission or upload mechanism	Mail-in return of device for data retrieval	Bluetooth between sensor and transmitter, cellular transmission between transmitter and server	In-house data download in clinic
Preliminary data processing, management and reporting	Medicare certified independent diagnostic testing facility, certified technician	Medicare certified independent diagnostic testing facility, certified technician	Clinic/Hospital-based technician
Weight (g)	34	50	Variable (average 100–150, min. 62)
Dimensions (mm)	123 × 53 × 10.7	160 × 60 × 15	100 × 60 × 25 (average)
Associated components	None	Wireless transmitter, battery charger	Leads, recorder, straps
Device cost^§^	Variable (US$329)^†^	Variable (US$718)^¶^	Variable (US$600 to $6000+)

*For other multi-lead, multi-channel ambulatory telemetry systems, please refer to a review by Mittal et al. (You et al., 2012)*.

* *Acquired by Medtronic plc*.

§ *Excluding clinic, technician and other fees*.

† *Direct self-pay price*.

¶ *Medicare negotiated price, qualified patient pays 20%*.

At a steady pace of miniaturization, long-term (months to years), “in-body” implantable devices such as the USB memory stick-sized (62 × 19 × 8 mm) Reveal® XT (Medtronic, Inc., Minnesota, USA and Ireland, EU) have also undergone a reduction in their footprint, with increased data storage capacity and longer duration of study (up to 3 years for Reveal® LINQ) (Figure 2). Similar to non-invasive devices, the injectable cardiac monitors (e.g., Reveal LINQ) are pushing the limits of minimally invasive cardiac monitoring. The associated costs as well as the suspect differential diagnoses will dictate the choice of non-invasive and invasive modalities of rhythm monitoring, given that the costs could differ by as much as 40-fold depending on the device (ranging from US$104 for short-term Holter monitor and US$275 for 30-day event recorder to US$4374 for implantable loop recorder for up to 2 years' deployment; prices may differ considerably depending on the institutions and countries) (Zimetbaum and Goldman, 2010).

## ECG patch devices

Adhesive AECG patch devices typically comprise a sensor system, a microelectronic circuit with recorder and memory storage, and an internal battery embedded in a relatively flexible synthetic matrix, resin, or other material. They are usually intended for medium-term use ranging from days to several weeks, depending on the device (Figure 1). The self-contained adherent unit typically has a low profile and can be affixed to the body surface, usually over the left upper chest area, by means of prefabricated adhesive material (Figure 2).

The main advantages of this kind of AECG system are that they are easy to use, leadless, minimally intrusive to daily activities, water-resistant, hygienic (i.e., single use only), and incur no upfront cost to the clinic for the initial device investment as compared to the wearable, reusable devices. Because of easy application of the adhesive AECG patch to skin and its unobtrusive maintenance-free nature, they have a high study completion rate (Shinbane et al., 2013), implying a high acceptance rate (long wear time) that should translate into improved compliance compared to other short- to medium-term devices such as the Holter monitor (Barrett et al., 2014).

Disadvantages of currently available adhesive AECG patch devices include their high cumulative consumer costs (due to non-reusability), dependence on the device company for raw data retrieval, the company technician's accurate collection and reporting of raw data, and generation of a summary report. As the ZIO XT system (iRhythm Technologies, Inc., San Francisco, California, USA) requires that the user return the device in a postage-paid envelope upon study completion, there is a lag time from postage to data retrieval and processing by the company's ZIO ECG Utilization Service (ZEUS®). The turnaround time from device submission to availability of a summary report could take days before the reader (usually, a cardiologist or cardiac electrophysiologist) has access to review the data and synthesize a diagnosis based on clinical grounds. The duration of this processing period may not be of concern when the ambulatory patient's underlying condition is felt to be relatively benign, without immediate hemodynamic consequence, or when the device is used to assess arrhythmia burden or rate control in response to suppression therapy. Some of these logistical issues are circumvented by the Corventis NUVANT/Medtronic SEEQ mobile cardiac telemetry (MCT) system (Engel et al., 2012) through real-time data transmission to the company's data network. However, availability of data is still dependent on time required for data processing by the company's Monitoring Center, though within a relatively shorter time frame compared with the ZIO system. The inherent two-piece design of the MCT system, consisting of the sensor (PiiX®) and the separate cellular data transmitter (zLink®), may be less convenient for some users. As an extension to the NUVANT MCT system, the AVIVO® mobile patient monitoring (MPM®) system features additional monitoring parameters including respiratory rate, heart rate variability, activity, posture, and fluid status. These available systems point to the need for innovative engineering to harness the strengths of each technology in order to provide a continuous wireless monitor that can not only record all beats and therefore provide arrhythmia burden, but can also wirelessly send important arrhythmic events in real time for timely diagnosis and treatment.

The ZIO Patch and the NUVANT/SEEQ MCT systems (Table 1) have been well accepted by study subjects, with 93.7% of patients finding the former comfortable and 81% indicating preference over the Holter monitor (Barrett et al., 2014). Depending on the patient's clinical presentation, the clinician's index of suspicion for a particular underlying arrhythmia or conduction system disease, the timeframe allowed for ECG data access, and taking into account the designs and logistical aspects inherent in each device, the ordering clinician will have to weigh these considerations when choosing the appropriate device. The ZIO XT, NUVANT/SEEQ MCT, and AVIVO MPM are approved by the U.S. Food and Drugs Administration (FDA) and have received the CE Mark for use in the European Union.

At the time of this writing, Medtronic plc. had acquired Corventis, Inc. and was in the process of relaunching the NUVANT MCT as the Medtronic SEEQ MCT for short- to medium-term cardiac monitoring to complement their long-term Reveal LINQ injectable monitor product line. Meanwhile, iRhythm Technologies, Inc. has evolved their ZIO System into a second-generation product, ZIO XT Patch, after successes with their first-generation ZIO Patch. As the demand for user-friendly adhesive AECG patch devices continues to increase, their availability from Philips (Ackermans et al., 2012) (Eindhoven, the Netherlands, EU) and other device manufacturers is expected in the near future.

## Arrhythmias and conduction system disorders detection by AECG systems and related devices

Clinically relevant arrhythmias and conduction system abnormalities that are detectable and reportable on AECG studies include sinus tachycardia, bradycardia, AF, AFL, supraventricular tachycardia, junctional rhythms/tachycardia, atrial and supraventricular ectopy (premature complexes), ventricular ectopy, ventricular tachycardia, pause (≥3 s), second-degree atrioventricular block (type I Wenckebach, type II Mobitz, high-grade AVB), and third-degree AVB (complete heart block) (Table 2). Although QT intervals (e.g., drug-induced), QT dispersion, and ST segment changes (e.g., myocardial ischemia) are not routinely reported, data analysis and results reporting could in theory incorporate these parameters for research purposes or for clinical studies requiring such customization. In particular, the limitations with ST segment monitoring reflect the lack of specificity and the inherent bias if the endpoints are gold-standard coronary angiography and intervention; there is currently no “hard” endpoint data such as myocardial infarction that is clinically relevant. Among clinically important and common arrhythmias, ventricular tachyarrhythmia and third-degree AVB are concerning for potential hemodynamic and circulatory compromise, whereas AF is the most common arrhythmia and strongly associates with stroke, increased risk of cardiomyopathy, congestive heart failure and death (Wolf et al., 1978; Krahn et al., 1995; Zipes, 1997; Chen et al., 2013; Reinier et al., 2014).

**Table 2:** **Overall organization of clinical and electrocardiographic data from ambulatory cardiac monitoring**.

**I. General**
a. Subject information
b. Enrollment period—days, hours
c. Analysis time—days, hours
d. Heart rate—maximum, minimum, range, average
e. Subject triggered events and diary entries
**II. Arrhythmia type, conduction system abnormalities and specifics**
a. Sinus tachycardia—number of episodes, duration, average rate, range
b. Bradycardia—number of episodes, duration, average rate, range
c. Pauses—number of episodes, duration, range
d. Junction rhythms or ectopy—burden (%), quantity
e. Atrioventricular block (type I, type II, 2:1, high-grade)—quantity
f. Complete heart block (third-degree)—quantity, duration
g. Atrial ectopy—burden (%), quantity
h. Atrial fibrillation—burden (%), range, rate, average
i. Atrial flutter—burden (%), range, rate, average
j. Supraventricular ectopy or tachycardia—burden (%), quantity
k. Wide complex tachycardia—quantity, rate
l. Ventricular ectopy (single, couplet, triplet, bigeminy, trigeminy)—type, burden (%), quantity
m. Ventricular tachycardia (≥3 beats)—sustained (≥30 s) or non-sustained (<30 s), burden (%)
**III. Other relevant information**
a. Subject triggered events relating to the above arrhythmias or conduction system abnormalities

Detection of paroxysmal AF is one of the main utilities of AECG monitoring. AF detection algorithms configured in ECG patch devices and data analysis software are usually proprietary, but methods based on the Lorenz plot (or its variants) of successive ventricular response (R–R) intervals are used to distinguish AF from normal sinus rhythm and/or other arrhythmias based on the premise of their different dispersion characteristics. The ability of the device to discriminate AF from atrial or other supraventricular tachycardia requires rigorous testing during device engineering and development, often using multiple test data sets [e.g., Massachusetts Institute of Technology-Beth Israel Hospital (MIT-BIH) database, IMPROVE database, American Heart Association (AHA) database)]. Test performance characteristics are compared against benchmarks. In the real-world setting, ECG patch devices (ZIO Patch) have very high concordance (*R* = 0.96) in detection of AF compared with Holter monitor (Rosenberg et al., 2013). When atrial or supraventricular tachyarrhythmia occurs for ≥30 s, the episode is by default recorded as an event by available AECG patch devices.

The definition of AF may, however, depend on the clinical study context. For example, some clinical studies only consider AF as being present when the episode lasts ≥30 s, a cut-off used to demarcate freedom from AF vs. AF recurrence in ablation studies (Calkins et al., 2012). In the TRENDS study, increased AF burden detected by pacemakers or defibrillators correlated with increased thromboembolic events (ischemic stroke and transient ischemic attack) (Glotzer et al., 2009), and more than 6 min of rapid atrial rate (or atrial tachyarrhythmia, a recognized precursor of AF, with atrial rate >190 beats per minute) correlated with increased risk of ischemic stroke or systemic embolism (Healey et al., 2012). Using pacemakers programmed to log rapid atrial rate, atrial tachyarrhythmia was also found to be an independent predictor of death in the MOde Selection Trial (MOST) study (Glotzer et al., 2003). The decision on whether to initiate anticoagulation is clinical, depending on the patient's risks (e.g., as gauged by risk scores such as CHADS_2_, CHA_2_DS_2_-VASc, and HAS-BLED), the duration and burden of AF, and other patient factors (Pisters et al., 2010; You et al., 2012; Lip, 2013).

The prevalence estimate of atrial arrhythmias is primarily dictated by the subject population under study. In the general elderly population the prevalence of AF is in excess of 10% (Heeringa et al., 2006), whereas an academic electrophysiology practice, for example, could be referred patients (mean age 56.7 years ±20.2) with high pre-test probability of AF/AFL and an actual prevalence of up to 20% (Eisenberg et al., 2014). The lifetime risks for AF at age 40 years is 26% for men and 23% for women based on risk calculations up to 95 years, and the heightened risks remain similar at age 80 years (22.7 and 21.6%, respectively) (Lloyd-Jones et al., 2004). Despite detailed reporting of risk estimation and AF incidence rates in various populations in Europe and North America (Krahn et al., 1995; Kannel et al., 1998; Heeringa et al., 2006), it is recognized that the majority of those large epidemiological studies were based on conventional chart review, physical exam and 10-s ECGs for diagnosis of AF (Wolf et al., 1978; Krahn et al., 1995; Kannel et al., 1998; Go et al., 2001; Lloyd-Jones et al., 2004; Heeringa et al., 2006; Dewland et al., 2013). Those case finding approaches are considered low-yield (Mittal et al., 2011) and most probably underestimated the true prevalence of AF. The ability to detect AF early is likely influenced by a surprisingly low prevalence of symptoms in this population. In a ZIO Patch study of 524 consecutive patients, 91 were identified with AF (Eisenberg et al., 2014). Only 46% of patients experienced symptoms during a mean follow-up of 7 days. Furthermore, patients with permanent AF were even less likely to report symptoms. Device deployment for an average of 7 days likely accounted for the high diagnostic yield in this patient population with a relatively high pre-test probability for an arrhythmia compared to the general population (Eisenberg et al., 2014).

The first diagnosis of AF often occurs in the unfortunate setting of an acute ischemic stroke (Sherman et al., 1984). In that patient population, it was clearly demonstrated that 24-h ECG monitoring was profoundly insufficient for diagnosing AF when compared to 72-h or 21-day monitoring (Schuchert et al., 1999; Tayal et al., 2008). In a study of 82 consecutive patients 2–3 weeks after an acute ischemic stroke and in whom resting ECGs showed normal sinus rhythm and no previously documented AF, only 1 patient (1.2%) was found to have paroxysmal AF within the first 24 h, 2 patients (2.4%) by 48 h and another 2 patients (2.4%) at 72 h (Schuchert et al., 1999). In another study of 56 patients with cryptogenic TIA or stroke using 21-day mobile telemetry monitoring, AF was diagnosed after a median of 7 days (Tayal et al., 2008). Moreover, 27 asymptomatic AF episodes were detected in 13 patients, of which 23 (85%) were <30 s and the remaining 4 (15%) were 4–24 h in duration (Tayal et al., 2008). These studies highlight the challenges in diagnosing AF with conventional monitoring even in relatively high arrhythmia burden patients with paroxysms, and support the use of prolonged ECG monitoring in most patients suspected to have atrial arrhythmia(s) and/or neurologic symptoms suggestive of impending or ongoing TIA or stroke that warrant close monitoring and aggressive cardiovascular risk modification. Using the 30-day event recorder or the Reveal XT implantable cardiac monitor in two independent studies of cryptogenic stroke (EMBRACE and CRYSTAL AF, respectively), their superiority over standard 24-h ECG monitoring for diagnosis of AF lasting 30 s or longer was strongly affirmed [16.1% vs. 3.2% by 90 days in EMBRACE (Gladstone et al., 2014); 8.9% vs. 1.4% AF diagnosed by 6 months and 12.4% vs. 2.0% by 12 months in CRYSTAL AF (Sanna et al., 2014)], irrespective of study design, patient demographics (e.g., mean age of 73 years in EMBRACE and 61 years in CRYSTAL AF) and study endpoints. Overall, this magnitude of increase in diagnostic yield is phenomenal. In the ASSERT study on 2580 patients with mean age of 76–77 years and in whom pacemaker implantation was indicated, device monitoring of subclinical atrial tachyarrhythmias that preceded the development of clinical AF was observed, shedding new light on the clinical epidemiology and natural history of the disease (Healey et al., 2012). These findings refute subclinical atrial tachyarrhythmias in elderly patients as simply benign.

Prolonged ECG monitoring studies have revealed that AF remains vastly under-diagnosed, and that duration of cardiac monitoring following acute ischemic stroke should be extended beyond 24–48 h (Schuchert et al., 1999; Tayal et al., 2008; Elijovich et al., 2009). With recent data affirming increased diagnostic yield using AECG patch devices (ZIO Patch and Corventis NUVANT MCT) compared to Holter monitors in patients with a relatively high pre-test probability for an arrhythmia (Rosenberg et al., 2013; Shinbane et al., 2013; Barrett et al., 2014), the epidemiology of AF in the general population warrants a reappraisal.

The use of AECG in the detection and surveillance of arrhythmias in patients with congenital heart disease is expanding. Arrhythmias remain the most frequent clinical sequelae for this patient population and are associated with increased risks of thromboembolic events (Jensen et al., 2015) and sudden cardiac death (Walsh, 2014). The prevalence of atrial arrhythmias among adults with congenital heart disease over a 22-year period is estimated at 15% (Bouchardy et al., 2009), and the 20-year risk of developing atrial arrhythmias is ~7% at 20 years of age, increasing to 38% at 50 years (Bouchardy et al., 2009). Severity of congenital heart disease also correlates with development of arrhythmias, with moderate to severe forms having significantly higher risks; however, even those with mild forms still remain at some risk (Walsh and Cecchin, 2007). Much of our current understanding of arrhythmias and conduction system disease in patients with congenital heart disease have come from studies using 12-lead ECG and Holter monitor (Glatz et al., 2010; Rodriguez et al., 2012; Czosek et al., 2013). Data on longer duration recording are lacking.

In the pediatric population, the quantity of AECG patch devices used remains low compared to that in adults. Nevertheless, ZIO patch data on 3209 consecutive children (mean age 12.5 years, range 1 month to 17 years) collected between 2011 and 2013 in a national registry suggested a relatively high diagnostic yield and short time to first detection of arrhythmias in this population (Bolourchi and Batra, 2015). Approximately 44–50% of the diagnoses were made beyond 48 h of cardiac monitoring, and the mean times to first detected and first symptom-triggered arrhythmias were 2.7 ± 3.0 and 3.3 ± 3.3 days, respectively (Bolourchi and Batra, 2015). The use of continuous ECG adhesive patch device requiring no upkeep or maintenance for prolonged periods of at least 1 week seems prudent particularly in children and young adults who have high pre-test probability for concerning arrhythmia.

## Clinical performance and evidence

Continuous, non-invasive AECG monitoring for up to 14 days can be provided by each ZIO device, and up to 7.5 days for each Medtronic SEEQ/Corventis NUVANT MCT (up to 30 days when four sensors are used serially). Since the time to first clinically relevant arrhythmia averages 5.8 ± 6.1 days (Shinbane et al., 2013), these systems have high likelihood of clinching a diagnosis where Holter monitors or event recorders (in case of an asymptomatic arrhythmia) fall short. Indeed, ZIO Patch use for ~10.8 days resulted in detection of 81% more arrhythmias compared with 24-h Holter monitoring (38 vs. 21 events, *P* < 0.001) in a study of 75 consecutive patients referred for AF management at a tertiary medical center (Rosenberg et al., 2013). In an analysis of 26,751 consecutive patients who wore the ZIO Patch for 48 h vs. the entire duration of 7.6 ± 3.6 days, the diagnostic yield significantly increased from 43.9 to 62.2% for any arrhythmia, and from 4.4 to 9.7% for symptomatic arrhythmia (Turakhia et al., 2013). Although there is a very high concordance between the ZIO Patch and Holter monitor in AF episode detection (*R* = 0.96) (Rosenberg et al., 2013), Cheung and colleagues pointed out that within the first 24 h of monitoring of any one of six arrhythmias (supraventricular tachycardia, AF/AFL, pause >3 s, AVB, ventricular tachycardia, or polymorphic ventricular tachycardia/fibrillation), the Holter monitor detected about 17% more reportable events (on average, 61 events by Holter vs. 52 events by ZIO Patch, *P* = 0.013) than the ZIO Patch (Cheung et al., 2014). This suggests that while little difference existed in the accuracy and consistency of AF detection between the ZIO Patch and Holter monitor, other arrhythmias and conduction disorders may have potentially been under-detected by the ZIO Patch, possibly owing to its single-channel nature compared with the multi-channel Holter monitor. The possibility of Holter monitor over-detecting arrhythmias and conduction disorders can be ascertained upon inspection of raw data. Differences in detection algorithms could also be explanatory. It would be essential for future studies to categorically identify the strengths and weaknesses for each patch device in detecting each type of arrhythmia and conduction system disease, to enable clinicians to choose the most appropriate device.

The ZIO Patch has been demonstrated to be useful in diagnosing and in guiding clinical management in different settings. In a multicenter study of 174 patients presenting to the emergency department with arrhythmia-related symptoms (palpitations, syncope and/or dizziness) who were subsequently discharged and enrolled into the study, the overall diagnostic yield was 63.2% for arrhythmias (27.6% supraventricular tachycardia ≥8 beats, 10.9% supraventricular tachycardia ≥4 but <8 beats, 8.1% ventricular tachycardia, 6.3% all AF, and the remaining comprised of other arrhythmias and conduction system disorders) (Schreiber et al., 2014). In another study of patients undergoing management of AF, the use of ZIO Patch led to a change in clinical management in over 28% of cases (Rosenberg et al., 2013). Moreover, study subjects found the ZIO patch more comfortable (Turakhia et al., 2013) and preferable than the Holter monitor (93.7% vs. 51.7%, respectively) (Barrett et al., 2014), and the total wear time of the NUVANT MCT was high (completing 90% of days prescribed in 715 subjects) (Shinbane et al., 2013), indicative of good compliance for both patch devices (Barrett et al., 2014).

## Clinical implications

Adhesive AECG patch devices have recently been demonstrated to be superior to Holter monitors in diagnosing AF, largely due to a longer study period and higher study completion rate owing to unobtrusive, user-friendly designs. They will continue to be useful tools for quantifying arrhythmia burden and surveillance of asymptomatic or symptomatic arrhythmias and conduction system diseases such as intermittent high-grade AVB or sick sinus syndrome. When used in large cohort studies, these devices may be invaluable for recharting the epidemiology of AF and other arrhythmias in both the general and ambulatory patient populations. Supported by evidence of practice-changing impact on clinical management (Rosenberg et al., 2013), the benefits of adhesive AECG patch devices over conventional short to medium term monitors should encourage their broader yet judicious use, particularly for paroxysmal atrial arrhythmias, documentation of burden of ventricular ectopy, and during continual outpatient workup of cryptogenic stroke when a suspect atrial arrhythmia evaded capture on inpatient telemetry. The application of adhesive AECG patch devices in children and in patients with congenital heart disease is growing, though data on diagnostic yield is still lacking. With longer wear period, the yield for detecting clinically significant arrhythmias and symptom-provoking arrhythmias and conduction system disease is expected to increase.

Available clinical society guidelines have yet to incorporate the use of AECG patch devices. These devices are currently being prescribed for similar indications as for Holter monitor, however, differences in device characteristics, study duration, diagnostic yield and study completion rate call for special attention in guideline development and revision. In the near future, the Heart Rhythm Society is expected to set out recommendations to improve device use appropriateness and aid management decisions.

## Vision of the future

Several years ago, Google (Google Health), Microsoft (Microsoft HealthVault), Dossia (Dossia, an open-source software supported by a large number of health industry stakeholders) and others initiated efforts to democratize digital health record stewardship using cloud-based platforms to enable easy access via the internet (Weitzman et al., 2009). Successes have thus far been hampered by privacy concerns and by the conventional, patriarchal model and proprietary silo-building inherent in established health care organizations. Internet-accessible PCHR have been championed by some, but many issues ranging from user's technological literacy to confidentiality and privacy risks have been voiced and remain areas of vigorous debate and interest (Weitzman et al., 2009). Moreover, there exists controversies over information access and appropriate usage by keyholders and other parties, data disclosure and privacy (Haas et al., 2011).

With emergence of AECG patch devices, smartphone-enabled wireless ECG and cardiac monitors over the past few years, the ease with which voluminous health data can be generated has accelerated. Revisiting the open-access PCHR models may lead to unfettered access and empower individuals to self-manage and potentially self-diagnose under physicians guidance. Notwithstanding the concerns of a loss of control in patient management, this modality of health information management along with well-planned, scalable digital health infrastructure could enable physicians to increase the volume of patients seen, reduce the time to diagnosis, improve efficiency and efficacy of disease management, and reduce unnecessary clinic visits and hospital admissions. At the forefront of digital health evolution, the above described miniaturized technologies coupled with PCHR are poised to improve patient adherence, and the detection, characterization and monitoring of cardiac arrhythmias—readily digitalized markers and phenotypes of cardiovascular disease—that are already making inroads into clinical diagnosis, patient management and healthcare transformation.

### Conflict of interest statement

EF, MRJ, RD, JS, SC, LG, PC, RS, and NP declare that there is no conflict of interest. HH is a recipient of a research grant from Medtronic, Inc.
